# Adherence to Iron and Folic Acid Supplementation and Its Associated Factors among Pregnant Women Attending Antenatal Care at Bwindi Community Hospital, Western Uganda

**DOI:** 10.1155/2021/6632463

**Published:** 2021-06-05

**Authors:** Christine Nimwesiga, Mereth Murezi, Ivan Mugisha Taremwa

**Affiliations:** ^1^Institute of Public Health and Management, Clarke International University, Kampala, Uganda; ^2^Uganda Nursing School, Bwindi-UCU Affiliate, Uganda; ^3^Institute of Allied Health Sciences, Clarke International University, Kampala, Uganda

## Abstract

**Methods:**

This was a cross-sectional study that used an interviewer-administered questionnaire and reviewed medical records. Binary and multivariable logistic regression analyses were used to identify factors associated with iron and folic acid supplementation. Adjusted odds ratio (AOR) with 95% confidence interval (CI) and *p* value < 0.05 were used to assess for statistical significance.

**Results:**

We enrolled 438 pregnant women aged 16 to 41years. Participants' mean age (±standard deviation (SD)) was 25.9 (±3.17) years. The self-reported adherence to iron and folic acid supplementation (consumed ≥4 tablets a week or 20 tablets in a month daily without missing the prescribed dosage) was 22.37% (*N* = 98). Among the adherent pregnant women, the reported reasons (and their respective proportionality) for adherence were getting advice and counseling from the healthcare worker about the good effects of iron and folic acid supplementation (*N* = 34, 34.69%) and knowledge about the health benefits of iron and folic acid supplementation such as preventing anemia (*N* = 16, 16.33%), among others. On the other hand, the reported reasons (and their respective proportionality) for iron and folic acid nonadherence were forgetfulness (*N* = 158, 46.47%), taking too many pills (*N* = 7, 2.06%), not knowing the usefulness of iron and folic acid supplementation (*N* = 29, 8.53%), fear of the side effects of the medication (*N* = 119, 35.00%), and not getting the supplement from the hospital (*N* = 27, 7.94%). Bivariable and multivariable logistic regression analyses indicated that pregnant women who were primigravida (adjusted odds ratio (AOR) = 4.5), who have parity of 2 or 3 (AOR = 3.4), who perceived importance of iron and folic acid supplementation to prevent anemia (AOR = 2.9), and who considered it important to take iron and folic acid supplementation (AOR = 2.9) showed a statistically significant association with adherence to iron and folic acid supplementation. Moreover, pregnant women who perceived the risk of not taking iron and folic acid supplementation (AOR = 5.2), those who received sufficient health education regarding the goals of iron and folic acid supplementation as well as the dangers of not taking the supplements (AOR = 4.4) and adequate counseling, and those who obtained an explanation of the effects of iron and folic acid (AOR = 4.8) showed a significant association with adherence to iron and folic acid supplementation.

**Conclusion:**

This study found a low adherence of iron and folic acid supplementation and was associated with obstetric and client- and health system-related characteristics. To this end, there is a need for individualized strategies targeting such factors and intensifying health education, guidance, and counseling to optimize adherence to iron and folic acid supplementation.

## 1. Background

Iron and folic acid deficiency is a widespread nutritional public health challenge among pregnant women [1, 2]. This is a result of increased body demand and poses severe consequences for both the productive and reproductive roles [2, 3], which may augment severe anemia in pregnancy and life-threatening congenital fetal anomalies such as neural tube defects, hemorrhagic newborn disease, and physical and cognitive dysfunctioning [4]. To avert these effects, iron and folic acid supplementation (IFAS) among pregnant women and optimal adherence have been recommended [1, 2, 5]. Overtime, IFAS has been ensured through varied strategies: firstly, the use of tablets with fixed dose combination, and secondly, the inclusion of iron and folic acid tablets in the list of essential drugs in the national drug formularies [6–8]. Despite these interventions, adherence to IFAS remains unacceptably low in Africa [9–12]. In Uganda, two research studies reported a low IFAS adherence among pregnant women, that is, 11.6% over a 30-day period among pregnant women attending Mulago National Referral Hospital (MNRH) [13] and 13.2% in Kiboga District [14]. The various determinants to the adherence of IFAS among pregnant women included knowledge of the pregnant woman in regard to iron and folic acid supplementation, gravidity, counseling offered especially on the management of its side effects, forgetfulness, travel, age, literacy, socioeconomic status, cost of tablets, perceived side effects, supplement stock-outs, and clarity on importance of iron and folic acid supplementation [5, 11–15]. The reported low level of adherence, coupled with the associated barriers, agitates the need to explore the factors associated with iron and folic acid supplementation adherence to improve the outcome of the current strategy. At Bwindi Community Hospital (BCH), despite the nationwide implementation of providing free iron/folate supplements to all pregnant women, the hospital records for five years (2014-2018) indicated an unacceptably very low iron/folic acid adherence (unpublished hospital report, 2018). At BCH, anecdotal evidence shows that adherence is at 38% and this poses a critical healthcare challenge. Although limited studies have been done to identify factors associated with low adherence, in BCH, there is no single study done to determine factors associated with low adherence to iron and folic acid supplementation, hence the need for this study. The objective of this study was to assess the adherence to iron and folic acid supplementation and the associated factors among pregnant women attending antenatal care at Bwindi Community Hospital, in Western Uganda.

## 2. Methods

### 2.1. Operational Definition

Adherence was considered for a pregnant woman who took at least 57% of the expected dose of IFAS in the previous week prior to the study, which is an equivalent of taking a single tablet daily for four days in the week consecutively or 20 tablets in a month without missing the prescribed dosage.

### 2.2. Study Design and Duration

The study used a cross-sectional study design, between the periods of August 2018 and February 2019.

### 2.3. Study Setting

The study was conducted at the ANC clinic of Bwindi Community Hospital (BCH). Bwindi Community Hospital is a Private Not for Profit (PNFP) facility founded in 2003 by the U.S. missionaries. It is located in Buhoma village, Mukono Parish, Kayonza Subcounty, Kinkiizi West Constituency, and Kanungu District in Western Uganda. The hospital has a bed capacity of 112 and offers varied healthcare services such as comprehensive emergency obstetric care, curative outpatient and inpatient services, family planning, preventive services, growth monitoring, and antenatal care. It serves as a referral for other health facilities within neighborhood and adjoining districts.

### 2.4. Study Population, Sample Size Estimation, and Sampling

These comprised pregnant women attending ANC, who were attending for at least the second time and were supplemented with iron and folic acid tablets a month prior to the data collection period. Also, the study focused on the current pregnancy to avoid the recall bias. The sample size was estimated using a single population proportion estimation formula, with the following assumptions: the proportion of iron and folic acid adherence was considered at 11.6% [14], 95% confidence interval, 5% allowable error, and a 10% nonresponse rate. A total of 438 pregnant women were enrolled. The study used a random sampling strategy to include 438 participants. Pregnant women were given information about the study and subsequently asked to participate. These were interviewed consecutively, as they were enrolled. Those who were very sick or had an obstetric emergence were not enrolled.

### 2.5. Study Variables, Data Collection Tools, and Procedure

**Figure 1:**
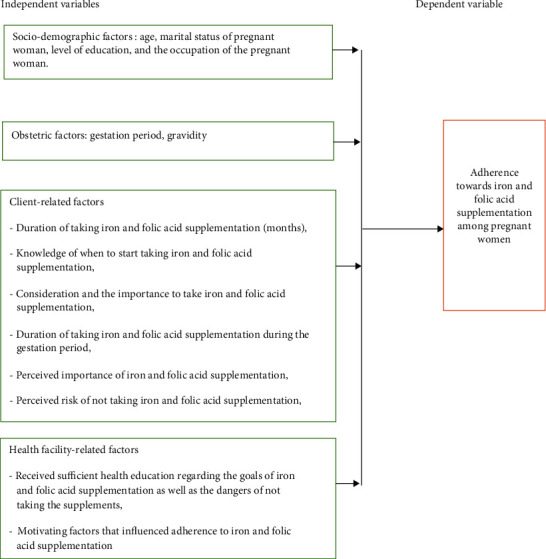
Conceptual framework developed from different literature [3, 9–15].

The dependent variable was adherence to iron and folic acid, while the independent variables were the sociodemographic and obstetric characteristics and client-related and health facility determinants of iron and folic acid supplementation adherence as given in Figure 1.

Data was collected using a pretested interviewer-administered structured questionnaire and ANC cards. The structured questionnaire was developed in English as guided by previous studies [3, 5, 9–15]. The questionnaire captured the sociodemographic and obstetric characteristics and client-related and health system determinants of iron and folic acid supplementation adherence. This was then translated into the local language (Runyankole-Rukiga) by a translator proficient in the language. The corresponding author crosschecked the translation, and modifications were made accordingly. The pretest of the questionnaire was carried out at Kisugu Health Centre IV on 22 (5.02%) of the pregnant women, and modifications were adopted as required to ensure clarity, wording, and logic flow.

Three members of the research team oversaw the data collection process and were supported by three research assistants who were trained midwives and had had a prior involvement in conducting research among pregnant women. For this study, a two-day training was conducted to enable understanding and practice of the data collection tools. The completed questionnaires were reviewed and crosschecked for completeness, accuracy, and consistency on a daily basis by the corresponding author who ensured data quality and compliance. Additionally, close daily supervision was ensured to monitor the performance of the research team and assistants and to deliver immediate corrective actions on mistakes noted. The completed and reviewed questionnaires were kept in a box file, in a lockable cupboard, and the key was only accessible to the study team or the corresponding author.

### 2.6. Data Management and Analysis

Data collected was entered into Epi Info version 7.2 and then exported into the Statistical Package for the Social Sciences (SPSS) version 24.0 for analysis. Descriptive statistics including tables and proportion were used to present the variables. Bivariable and multivariable logistic regression analyses were used to determine the association between dependent and independent variables. Variables with a *p* value of <0.20 during a bivariable analysis were incorporated into the multivariable logistic regression to control the possible effects of confounders. The analysis used the Hosmer and Lemeshow's goodness-of-fit test with a large *p* value (*p* > 0.05) to check the good fitness. Also, multicollinearity and confounding effect were checked by using a standard error. The variable without multicollinearity was considered for a multivariable model. The adjusted odds ratio (AOR) with corresponding 95% confidence interval (CI) was computed to see the strength of the association, and a *p* value of <0.05 was considered statistically significant.

### 2.7. Ethical Considerations

Ethical approval was obtained from the Research Ethics Committee of Clarke International University, after which administrative permission to carry out the study at Bwindi Community Hospital was obtained from the executive director. The study obtained written informed consent from all the participants who were ≥18years. Participants under 18 years signed a consent form as they are regarded as emancipated minors. The consent process was carried out in privacy, and no incentives were given. The study did not seek consent from a parent or legal guardian on behalf of the participants under the age of 18 years as this was not mandatory as per the guidelines on emancipated minors given in the Uganda National Council for Science and Technology (UNCST). Prior to consent, the study was introduced as part of the directed health talk to the antenatal care attendees. Additionally, anonymity of the participants was ensured at all stages of data analysis by excluding personal identifiers.

## 3. Results

### 3.1. Sociodemographic and Obstetric Characteristics of Study Participants

Four hundred and thirty-eight pregnant women aged 16-41years were enrolled. The mean ± standard deviation (SD) age of respondents was 25.9 ± 3.17 years. The majority of the study participants (65.07%, *N* = 285) were in the age group of 20–29 years, 42.24% (*N* = 185) had completed primary level education, and 84% were married. Also, most of the participants (50%) were gravida 2 or 3, and 53.20% (*N* = 233) were in their third trimester of pregnancy, as given in Table 1.

### 3.2. Adherence to Iron and Folic Acid Supplementation

Of the 438 pregnant women, the self-reported adherence to IFAS (consumed ≥4 tablets a week or 20 tablets in a month daily without missing the prescribed dosage) was 22.37% (*N* = 98). Relatedly, 77.62% (*N* = 340) of the pregnant women had not taken 4 or more tablets of IFAS per week in the past 1 month preceding the study. Among the adherent pregnant women, the reported reasons (and their respective proportionality) for adherence were getting advice and counseling from the healthcare worker about the good effects of iron and folic acid supplementation (*N* = 34, 34.69%) and knowledge about the health benefits of iron and folic acid supplementation such as preventing anemia (*N* = 16, 16.33%) among others. On the other hand, the reported reasons (and their respective proportionality) for iron and folic acid nonadherence were forgetfulness (*N* = 158, 46.47%), taking too many pills (*N* = 7, 2.06%), not knowing the usefulness of IFAS (*N* = 29, 8.53%), fear of the side effects of the medication (*N* = 119, 35.00%), and not getting the supplement from the hospital (*N* = 27, 7.94%).

### 3.3. Factors Associated with Iron and Folic Acid Supplementation

Bivariable and multivariable logistic regression analyses indicated that pregnant women who were primigravida (adjusted odds ratio (AOR) = 4.5, 95%confidence interval (CI) = 2.21-4.73), those whose parity was 2 or 3 (AOR = 3.4, 95%CI = 2.73-4.02), those who considered it important to take IFAS (AOR = 2.9, 95%CI = 2.39-3.40), those who perceived the importance of iron and folic acid supplementation to prevent anemia (AOR = 2.9, 95%CI = 2.39-3.40), those who perceived it as a risk not to take iron and folic acid supplementation (AOR = 5.2, 95%CI = 4.06-6.18), those who received sufficient health education regarding the goals of iron and folic acid supplementation as well as the dangers of not taking the supplements (AOR = 4.4, 95%CI = 3.06-5.12) and adequate counseling (AOR = 3.6, 95%CI = 2.16-4.50), and those who obtained an explanation of the effects of IFAS (AOR = 4.8, 95%CI = 3.08-6.42) showed a significant association with adherence to IFAS after adjusting and controlling for all other variables at *p* value of <0.05.

The analysis indicated that pregnant women who were primigravida were 4.5 times more likely to adhere to IFAS as compared to those who were multigravidae (AOR = 4.5, 95%CI = 2.21-4.73). Also, pregnant women whose parity was 2 or 3 were 3.4 more likely to take four or more iron and folic acid tablets (AOR = 3.4, 95%CI = 2.73-4.02). Moreover, pregnant women who considered it important to take IFAS increased the odds of iron and folic acid adherence (AOR = 2.9, 95%CI = 2.39-3.40). Further, there was a 2.9 times likelihood of adhering to IFAS among those who perceived its importance to prevent anemia (AOR = 2.9, 95%CI = 2.39-3.40). Relatedly, pregnant women who perceived it as a risk not to take IFAS were 5.2 times more likely to adhere (AOR = 5.2, 95%CI = 4.06-6.18). Similarly, pregnant women who received sufficient health education regarding the goals of IFAS as well as the dangers of not taking the supplements were 4.4 times more likely to adhere (AOR = 4.4, 95%CI = 3.06-5.12). Pregnant women who received adequate counseling were 3.6 times more likely to adhere to IFAS (AOR = 3.6, 95%CI = 2.16-4.50), whereas pregnant women who obtained an explanation of the effects of IFAS presented with 4.8 times more likelihood of adhering (AOR = 4.8, 95%CI = 3.08-6.42), as shown in Table 2.

## 4. Discussion

Four hundred and thirty-eight pregnant women aged 16-41 years were enrolled. Of these, the self-reported adherence to IFAS was 22.37%. This value is lower than 55.5% that was reported from Debre Markos Town in Ethiopia [9] and 59.8% obtained from a study conducted in Assela Town, Ethiopia [16]. Additionally, this adherence is lower compared to 64.7% reported from Eritrea [17] and 70.6% from Mizan Aman Town in Ethiopia [15]. The observed low adherence is multifaceted and is in part attributed to the differences in the sociocultural differences and lower literacy rates in the current study setting as up to 47.94% of the studied pregnant women had not attained any formal education at all or had completed up to primary level of education. This is imperative; however, it ought to be recognized that educated women can understand the messages passed from healthcare providers and be put into practicing health messages [18–20]. On the other hand, the obtained adherence was higher than 11.6% reported among pregnant women attending Mulago National Referral Hospital (MNRH) [13], and 13.2% in Kiboga District [14]. The discrepancy in the obtained values is in part attributed to numerous factors related to the study participants and setting.

As it emerged among the adherent pregnant women, the reported reasons for adherence were getting advice and counseling from the healthcare worker about the good effects of IFAS and knowledge about the health benefits of IFAS such as preventing anemia. These reasons had earlier been reported [5, 21–23] and have been described as key determinants to the antenatal care package. On the other hand, the reported reasons for IFAS nonadherence were forgetfulness, taking too many pills, not knowing the usefulness of iron and folic acid supplementation, fear of the side effects of the medication, and not getting the supplement from the hospital [9, 24–26]. Similar to previous reports, forgetfulness of a drug has been a key determinant to bad medication adherence. For example, a study that was done in one of the areas in Uganda indicated that 82% of the pregnant women ever miss to take iron supplements due to forgetting [13]. Relatedly, a study conducted from South Africa indicated that forgetting to take the iron and folic acid supplements was due to busy schedules leading to forgetting [27]. The forgetfulness among the participants could be because being in rural area and with the majority being unemployed, they probably spend most of their time in gardens or become busy with casual labor and forget to carry with them their pills. The practice of too many drugs prescribed at the same time (polypharmacy) has been highlighted as an adherence barrier as supported by the previous research evidence [28, 29]. Polypharmacy was linked to nonadherence because pregnant women experienced medicine fatigue and suffered from frustration of many tablet intakes [30, 31]. Also, not knowing the usefulness of iron and folic acid supplementation was indicated as a hindrance to adherence. Consistent with previous studies, lack of awareness on importance of taking the medication ought to be emphasized among pregnant women [32, 33]. Also, the World Health Organization (2015) emphasizes the need to highlight that all the tablets are necessary to ensure good fetal outcome [4]. These factors negatively affected iron and folic acid supplementation and necessitate a critical consideration to improve the uptake.

The other reason for nonadherence was the fear of the side effects of the iron and folic acid supplementation. As previously explored, fear of having a big baby and related side effects of iron and folic acid supplementation have prevented adherence efforts [4]. Also, a study conducted from South Africa indicated that folic acid and ferrous sulphate made pregnant women feel nauseous, inclined them to vomit, and also caused gastrointestinal upset [27]. This was similar to a study report from the North Western Zone of Tigray, Ethiopia [34]. Besides the listed reasons for nonadherence to iron and folic acid supplementation, not getting the supplement from the hospital was highlighted as a major setback. This collaborates well with previous reports and highlights the negative attributes of drug stock-out in health facilities as some pregnant women may not comply with clinic purchases due to lack of money, as well as exorbitant charges [35–37]. Thus, to improve on the uptake of iron and folic acid supplementation, it is desirable to ensure regular hospital stocks of iron and folic acid tablets and to emphasize their correct use by the pregnant women.

The bivariable and multivariable logistic regression analyses indicated that pregnant women who were primigravida, had parity of 2 or 3, considered it important to take iron and folic acid supplementation, perceived the importance of iron and folic acid supplementation to prevent anemia, and perceived the risk of not taking iron and folic acid supplementation showed a significant association with adherence to iron and folic acid supplementation. In addition, pregnant women who received sufficient health education regarding the goals of iron and folic acid supplementation as well as the dangers of not taking the supplements and adequate counseling and pregnant women who obtained an explanation of the effects of iron and folic acid supplementation showed a significant association with adherence to iron and folic acid supplementation. Primigravida and low parity number (<4) are generally considered factors of adherence to the pregnancy requirements [38]. This is in agreement with previous studies which showed that young parity was associated with compliance [5, 39]; however, it contravenes previous evidence that primigravida and low parity risked missing iron and folic acid supplementation due to lack of knowledge and understanding towards care for their pregnancies [40]. This finding highlights the need to cautiously emphasize the strict need for pregnant women to adhere to iron and folic acid supplementation to ensure the best maternal and fetal outcome [41]. The other factors were the consideration of the importance of IFAS, the perception of the importance of IFAS to prevent anemia during pregnancy, and the perception of risk of not taking IFAS. These factors have been widely studied, and they affirm the importance of pregnant women understanding the positive attributes of IFAS [12, 16, 42]. These factors are as well supported by other research findings [5, 12, 16] and highlight a potential improvement in the uptake of iron and folic acid supplementation in pregnant women to know anemia and its consequences towards the pregnancy outcome. The current study also found that women who received sufficient health education regarding the goals of IFAS and the dangers of not taking the supplements and adequate counseling and pregnant women who obtained an explanation of the effects of IFAS had better adherence than those who did not receive. Similar findings were reported from earlier reports [5, 9, 11, 12]. This suggests that such explanations offer the opportunity to understand the purpose, importance, possible side effects and duration, dosage of the supplement, and tolerance to the associated adverse effects. Also, understanding the dangers of not taking the supplements is critical as pregnant women ensures adherence to subvert the likely consequences. Studies show that lack of health education on IFAS among pregnant women has greatly affected the adherence to IFAS [5, 12, 40, 42], necessitating targeted and focused information, training, and counseling on the importance of IFAS during pregnancy [43]. The understaffing, late coming of mothers, and workload could be contributing to nonhealth education of mothers which are mentioned to contribute to low adherence among participants in this study area. This study finding is similar to previous reports [44, 45] and highlights the need for concerted sensitization for behaviour change. Contrary to previous studies [11, 12], this study did not find a significant statistical association with the sociodemographic variables.

The findings of this study ought to be interpreted in light of the following study limitations: firstly, the 7-day compliance period is short and may not represent compliance throughout the pregnancy period. This period was utilized to minimize recall bias, and this has cross-sectional study design and is guided by previous studies. Also, the estimation of IFAS adherence by the self-report method may underestimate the prevalence of nonadherence when compared with objective measures like pill counts or biological assay medication adherence measures. Secondly, there may have been recall bias and subjectivity because the study heavily relied on verbal reports from the interviewees. Thirdly, the current study was conducted entirely within one center; differences of geographic location were not assessed, and this limits the generalization of study results.

## 5. Conclusions

This study found a low adherence (22.37%) to iron and folic acid supplementation and was associated with various obstetric and client- and health system-related characteristics. Adherence was linked to advice and counseling to pregnant women and perceived health benefits of iron and folic acid supplementation. On the other hand, numerous reasons for iron and folic acid nonadherence were reported and negatively affected the compliance to antenatal care recommendations. Bivariable and multivariable logistic regression analyses indicated obstetric and client- and health facility-related factors to assess the rate of adherence to IFAS. Based on these, the following recommendations have been proposed: (a) there is a need to increase health education on the goals for IFAS and guidance on how to swallow the drugs as recommended to increase adherence to IFAS, (b) there should be continuous sensitization of the community about the goals and benefits of IFAS, and (c) the community and other local leaders ought to continuously advocate and support early attendance of ANC by pregnant mothers and support mothers on IFAS to enable them finish the required doses.

## Figures and Tables

**Table 1:** Sociodemographic and obstetric characteristics of participants (*n* = 438).

Variables	Frequency (*N*)	Percentage (%)
Age (years)		
≤18	16	3.65
19-29	285	65.07
≥30	137	31.28
Marital status		
Married	368	84.02
Single	70	15.98
Level of education		
No formal education	25	5.71
Primary	185	42.24
Secondary	126	28.77
Tertiary	102	23.29
Occupation		
Employed	134	30.59
Unemployed	304	69.41
Gravidity		
Primigravida	131	29.91
2^nd^-3^rd^	219	50.00
≥4^th^	88	20.09
Parity		
0	34	7.76
1-2	174	39.73
3-4	230	52.51
Gestation period		
1^st^ trimester	115	26.26
2^nd^ trimester	90	20.25
3^rd^ trimester	233	5.20

**Table 2:** Factors associated with iron and folic acid supplementation among pregnant women.

Variables	Adherence		Crude odds ratio (95% CI)	Adjusted odds ratio (95% CI)	*p* value
	Yes, number (%)	No, number (%)			
Sociodemographic characteristics					
Age (years)					
≤18	7 (7.14)	9 (2.65)	1.26 (0.91-2.62)	1.03 (0.77-2.17)	0.215
19-29	59 (60.20)	226 (66.47)	1.13 (0.48-1.86)	1.02 (0.43-1.59)	0.166
≥30	32 (32.65)	105 (30.88)	1	1	
Marital status					
Married	84 (85.71)	284 (83.53)	1.38 (0.94-1.81)	1.17 (0.84-1.49)	0.312
Single	14 (14.29)	56 (16.47)	1	1	
Level of education					
No formal education	11 (11.22)	14 (4.12)	1.20 (0.69-2.16)	1.10 (0.62-2.01)	0.395
Primary	21 (21.43)	164 (48.24)	1.62 (0. 88-2.31)	1.23 (0.76-2.12)	0.098
Secondary	32 (32.65)	94 (27.65)	1.94 (0.96-2.47)	1.66 (0.79-2.21)	0.168
Tertiary	34 (34.69)	68 (20.00)	1	1	
Occupation					
Employed	43 (43.88)	91 (26.76)	1.6 (0.99-2.31)	1.4 (0.72-2.01)	0.114
Unemployed	55 (56.12)	249 (73.24)	1	1	
Obstetric characteristics					
Gravidity					
Primigravida	29 (29.59)	102 (30.00)	4.7 (2.67-5.11)	4.5 (2.21-4.73)	0.002^∗^
2^nd^-3^rd^	41 (41.84)	178 (52.35)	1.6 (1.27-2.01)	1.3 (1.19-1.87)	0.712
≥4^th^	28 (28.57)	60 (17.65)	1		
Parity					
1-2	53 (54.08)	121 (35.59)	3.9 (2.81-4.17)	3.4 (2.73-4.02)	0.003^∗^
3-4	45 (45.92)	219 (64.41)	1	1	
Gestation period					
1^st^ trimester	27 (27.55)	88 (25.88)	1.4 (0.93-1.67)	1.21 (0.74-1.56)	0.716
2^nd^ trimester	44 (44.90)	46 (13.53)	1.7 (0.81-2.2)	1.4 (0.74-2.06)	0.081
3^rd^ trimester	27 (27.55)	206 (60.59)	1	1	
Client-related characteristics					
Duration of taking iron and folic acid supplementation (months)					
1-3	38 (38.78)	211 (62.06)	1.1 (0.43-1.47)	1.08 (0.38-1.38)	0.257
4-6	46 (46.94)	74 (21.76)	1.4 (0.61-1.88)	1.2 (0.54-1.62)	0.377
≥7	14 (14.29)	55 (16.18)	1	1	
Knowledge of when to start taking iron and folic acid supplementation					
Immediately after realising I am pregnant	54 (55.10)	141 (41.47)	1.7 (1.44-2.31)	1.4 (0.96-1.88)	0.094
After 3 months	38 (38.78)	183 (53.82)	1.9 (1.63-2.33)	1.6 (1.42-2.21)	0.791
I do not know	6 (6.12)	16 (4.71)	1	1	
Considered it important to take iron and folic acid supplementation					
Yes	79 (80.61)	284 (83.53)	3.1 (2.49-3.51)	2.9 (2.39-3.40)	0.002^∗^
No	19 (19.39)	56 (16.47)	1	1	
Duration of taking iron and folic acid supplementation during the gestation period					
Throughout pregnancy	37 (37.76)	34 (10.00)	1.9 (1.71-2.26)	1.6 (1.32-2.13)	0.683
<9 months	59 (60.20)	297 (87.35)	1.3 (1.01-1.83)	1.1 (0.91-1.48)	0.417
I do not know	2 (2.04)	9 (2.65)	1	1	
Perceived importance of iron and folic acid supplementation					
Prevention of anemia	61 (62.24)	113 (33.24)	6.7 (5.80-7.38)	6.2 (5.41-7.17)	0.003^∗^
Good for the baby's growth and health	29 (29.59)	94 (27.65)	1.9 (0.71-2.21)	1.6 (0.62-2.09)	0.455
Prevents complications during delivery	6 (6.12)	46 (13.53)	1.4 (0.98-1.81)	1.1 (0.81-1.57)	0.391
Others	2 (2.04)	87 (25.59)	1	1	
Perceived as a risk not to take iron and folic acid supplementation					
Yes	69 (70.41)	191 (56.18)	5.5 (4.12-6.17)	5.2 (4.06-6.18)	0.002^∗^
No	29 (29.59)	149 (43.82)	1	1	
Days that iron and folic acid supplementation was taken in the previous 1 week					
<4	0 (0.00)	340 (100.00)	1.1 (0.61-1.73)	1.0 (0.55-1.24)	0.741
≥4	98 (100.00)	0 (0.00)	1	1	
Health facility-related characteristics					
Received sufficient health education regarding the goals of iron and folic acid supplementation as well as the dangers of not taking the supplements					
Yes	76 (77.55)	29 (8.53)	4.8 (3.18-5.22)	4.4 (3.06-5.12)	0.001∗
No	22 (22.45)	311 (91.47)	1	1	
Motivating factors that influenced adherence to iron and folic acid supplementation					
Availability of iron and folic acid at the hospital	47 (47.96)	27 (7.94)	1.9 (0.19-2.64)	1.4 (0.66-2.14)	0.527
Adequate counseling	29 (29.59)	33 (9.71)	3.9 (2.51-4.71)	3.6 (2.16-4.50)	≤0.001^∗^
Explanation of the effects of iron and folic acid supplementation	13 (13.27)	206 (60.59)	5.1 (3.11-6.98)	4.8 (3.08-6.42)	0.002^∗^
Clear instructions for the use of iron and folic acid supplementation	9 (9.18)	107 (31.47)	1	1	

95% CI denotes 95% confidence interval. ∗ denotes variables with a statistical significance.

## Data Availability

The datasets used and/or analyzed during the current study are available from the corresponding author on reasonable request.

## Abbreviations

AIDS: Acquired immune deficiency syndrome
ANC: Antenatal care
BCH: Bwindi Community Hospital
Hb: Hemoglobin
HIV: Human immunodeficiency virus
HMIS: Health management information system
IFAS: Iron and folic acid supplementation
WHO: World Health Organization.
